# Systematic Review and Meta-Analysis on the Association between IL-1B Polymorphisms and Cancer Risk

**DOI:** 10.1371/journal.pone.0063654

**Published:** 2013-05-21

**Authors:** Jiali Xu, Zhiqiang Yin, Songyu Cao, Wen Gao, Lingxiang Liu, Yongmei Yin, Ping Liu, Yongqian Shu

**Affiliations:** 1 Department of Oncology, The First Affiliated Hospital of Nanjing Medical University, Nanjing, China; 2 Department of Dermatology, The First Affiliated Hospital of Nanjing Medical University, Nanjing, China; 3 Department of Epidemiology and Biostatistics, MOE Key Laboratory of Modern Toxicology, School of Public Health, Nanjing Medical University, Nanjing, China; Centro di Riferimento Oncologico, IRCCS National Cancer Institute, Italy

## Abstract

**Background:**

Interleukin-1 beta (IL-1β), a pro-inflammatory cytokine, is emerging as a key mediator of carcinogenesis that characterizes host-environment interactions. Epidemiological studies investigating the association between two polymorphisms of IL-1B (−511C/T and +3954C/T) and cancer susceptibility have shown conflicting results. The aim of this study is to derive a more precise estimation of the relationship.

**Methods:**

Related studies were identified through a systematic literature search of PubMed and Web of Science from their inception to September 15, 2012. Summary odds ratios (ORs) and 95% confidence intervals (CIs) for the IL-1B −511C/T and +3954C/T polymorphisms and cancer risk were calculated. Heterogeneity among studies and publication bias were also tested.

**Results:**

The meta-analysis included 91 case-control studies in 85 publications, 81 studies for the −511C/T (19547 cases and 23935 controls) and 26 studies for the +3954C/T polymorphisms (8083 cases and 9183). The pooled results indicated that IL-1B +3954C/T (dominant model: OR = 1.15, 95% CI: 1.01–1.30) was significantly associated with increased overall cancer risk, especially among hospital-based case-control studies (dominant model: OR = 1.30, 95% CI: 1.02–1.66). As for −511C/T, we observed an inverse relationship in cervical cancer (dominant model: OR = 1.74, 95% CI: 1.35–2.23) and hepatocellular carcinoma (dominant model: OR = 0.68, 95% CI: 0.47–0.99). Moreover, −511C/T was associated with risk of specific subtypes of gastric carcinoma.

**Conclusion:**

This meta-analysis suggested that both the IL-1B –511C/T and +3954C/T polymorphisms might modulate cancer susceptibility. Further well-designed studies based on larger sample sizes should be performed to confirm the findings.

## Introduction

Cancer is considered to be a complex, multistep and fatal disease that results from interactions between environmental and genetic factors [1]. It is increasingly recognized that inflammation contributes to pathogenesis of many cancers [2], [3]. Chronic inflammation may result in oxidative stress and potentiate tumor promotion and progression [4]–[7]. Cytokines are glycoproteins or soluble proteins that act as mediators of inflammatory response and are integral to the function of immune cells. The role of cytokines in cancer immunity and carcinogenesis in general has been well established [8], [9]. They are aberrantly produced by tumor cells, macrophages and other phagocytic cells [10], [11]. These cytokines then activate transcription factors such as NF-κB, AP-1 and STAT3, thus inducing genes that stimulate cell proliferation and survival. In addition to enhancing the proliferation of mutated cells, an inflammatory microenvironment can also increase DNA mutation rates [12]. On the other hand, immune cells affect malignant cells through the production of cytokines, chemokines, reactive oxygen and others [11]. Thus, cytokines are particularly important in neoplastic initiation.

Interleukin-1 (IL-1) is a pro-inflammatory cytokine with multiple biological effects [13]. The IL-1 gene family on chromosome 2q13-14 encodes three proteins: IL-1α, IL-1β and their naturally occurring inhibitor IL-1RN. IL-1β, mainly produced by blood monocytes and tissue macrophages, has been implicated in mediating both acute and chronic inflammation [14]. Moreover, its property of stimulating the tumor microenvironment in favor of increased cell proliferation and tissue angiogenesis has been given much attention [15]–[18]. IL-1β is emerging as a key mediator of carcinogenesis that characterizes host-environment interactions.

The IL-1B gene is highly polymorphic and base transitions between C and T at positions –511 (C-T; dbSNP: rs16944), –31 (T-C; dbSNP: rs1143627) and +3954 (C-T; dbSNP: rs1143634) base pairs from the transcriptional site have been widely reported. In particular, the first two polymorphisms are located in the promoter region, and show high linkage disequilibrium [19], [20]. The IL-1B –31T/C substitution causes disruption of a TATA-box motif and has been found to markedly affect the binding affinity of several transcription factors [19], [21], [22] and thereby affect the transcription activity of IL-1B [21]. The IL-1B +3954 C/T in exon 5 is a synonymous single nucleotide polymorphism (SNP). In vitro studies have shown that both −511T and +3954T are associated with increased IL-1β secretion from lipopolysaccharide (LPS)-induced IL-1β protein secretion [23]–[25].

It is not surprising that these functionally important polymorphisms in the IL-1B gene might be associated with cancer susceptibility. Recently, a meta-analysis of IL-1B –31T/C polymorphism and cancer risk has suggested that the –31C allele is a low-penetrance protective factor for the development of cancer [26]. Furthermore, numerous epidemiological studies have investigated the association between IL-1B –511C/T and +3954C/T and different cancers, such as gastric, lung and breast cancers. However, the results remain inconsistent and inconclusive. A clearer understanding of the relationship between these two SNPs and cancer susceptibility is of clinical significance. In this report, a meta-analysis was conducted to provide an overview of all the relevant studies and synthesize conclusions on the associations between the IL-1B –511C/T, +3954C/T polymorphisms and cancer susceptibility.

## Materials and Methods

### Literature Search Strategy

Electronic databases (PubMed and Web of Science) were comprehensively searched using combinations of the terms “interleuk-1/IL-1” or “Interleuk-1B/IL-1B/IL-1 beta”, “polymorphism” and “cancer” or “tumor” (the last search update on September 15, 2012). For each identified study, additional studies were sought from its citations, references and from the database option “Related Articles”.

### Inclusion and Exclusion Criteria

Eligible studies were selected according to the following explicit inclusion criteria: (i) case-control study evaluating the association between at least one of the two polymorphisms (IL-1B −511C/T and +3954C/T) and cancer susceptibility, (ii) sufficient genotype data presented to calculated the odds ratios (ORs) and 95% confidence intervals (CIs). Major reasons for exclusion of studies were: (i) only cancer group, (ii) no usable genotype frequency data, (iii) duplicate of earlier publication, (iv) publication not in English.

### Data Extraction and Quality Assessment

Two investigators (Jiali Xu and Zhiqiang Yin) extracted information from all eligible publications independently according to the criteria listed above. The following information was gathered from each study: the first author’s name, year of publication, cancer type, country of origin, ethnicity of subjects, source of control, genotyping method, number of cases and controls and genotype frequency (Table S1). The quality assessment of each study was carried out independently by the two investigators using the Newcastle-Ottawa Scale (NOS) [27]. Studies with a score equal to or higher than 5 were considered “high-quality”, whereas those scored less than 4 were considered “low-quality”. All item-specific ambiguities were discussed by investigators’ consultation until consensus was achieved.

### Statistical Analysis

We first calculated Hardy–Weinberg equilibrium (HWE) in the controls for each included study using a goodness-of-fit test and for which *P*<0.05 was considered not compliant with HWE. The strength of the association between the two SNPs (IL-1B −511C/T and +3954C/T) and cancer risk was measured by ORs with 95% CIs. Pooled ORs were obtained from combination of single studies by homozygote comparison (TT vs. CC), heterozygote comparison (CT vs. CC), dominant model (TT+CT vs. CC) and recessive model (CC+CT vs. TT), respectively. The heterogeneity among different studies was checked by the Q-test [28]. If the *P* value is <0.10, a random effect model with the DerSimonian and Laird method was used to pool the results. Otherwise, a fixed-effect model with the Mantel-Haenszel method was then used [29].

Sensitivity analysis was performed to assess the stability of the results by omitting a single study in this meta-analysis each time to reflect the influence of individual data on the pooled OR. To further explore the potential source of heterogeneity among studies and test the effects of study characteristics on the overall estimates, subgroup analyses and meta regression were performed stratified by cancer types (if one cancer type contained fewer than three individual studies, it was grouped into the “other cancers”), ethnicity (Caucasian, Asian or Others; Others included African and Mixed populations), and source of controls (population-based or hospital-based).

Publication bias was diagnosed with Begg’s funnel plot and Egger’s linear regression method [30]. Asymmetric or incomplete funnel shaped plots and *P*<0.05 in Egger’s test indicated the presence of potential publication bias. All statistical analyses used STATA 12.0 (STATA Corp, College Station, Texas). Except for heterogeneity statistics (where significance was declared if *P*<0.10), each *P*-value <0.05 was considered to be statistically significant. All *P*-values were 2-sided.

## Results

### Flow of Included Studies

**Figure 1:**
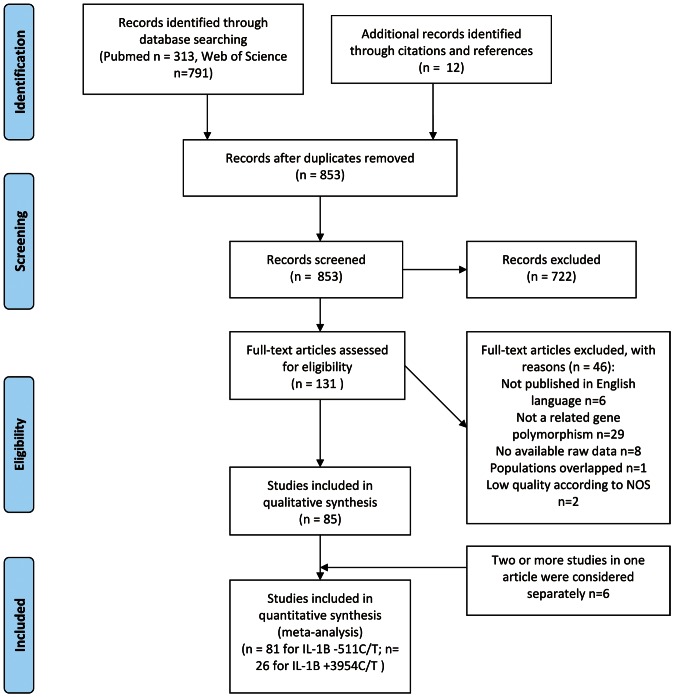
Flow chart of study selection.

Figure 1 depicts the results of the literature search. After review of abstracts, 131 full-text potentially relevant publications were retrieved to be assessed for eligibility. Among the 46 full-text articles excluded, one article [20] was updated by a new publication [31], and two case-control studies were considered “low-quality” (score = 3 and 4) using the NOS quality assessment instrument and thus were excluded [32], [33]. By contrast, five articles each mentioned two or more independent case-control studies, and studies included in these articles were treated as separate studies [34]–[38]. Finally, a total of 91 case-control studies in 85 publications, of which there were 81 studies for the −511C/T polymorphism [19], [31], [35], [37]–[83] [34], [36], [84]–[106] and 26 studies for the +3954C/T polymorphism [19], [34], [36], [42], [50], [51], [54], [56], [61], [65], [74], [81], [82], [105], [107]–[116], were included in the meta-analysis based on our search strategy and eligibility criteria.

### Study Characteristics

Detailed characteristics of the aggregated data for 91 case-control studies are summarized in Table S1. The vast majority of the included studies – all except 14 studies for –511C/T and 2 studies for +3954C/T – indicated that the genotype distributions in the controls were consistent with HWE. There were 47 studies of subjects of Asian descent, 32 studies of subjects of Caucasian descent and 12 studies of subjects with ethnicity “Others” (7 mixed-ethnicity studies and 3 African studies). Minor allele frequencies (MAFs) of −511C/T and +3954C/T of controls in different populations are graphed as Figure S1.

### Quantitative Data Synthesis

**Association of the IL-1B –511C/T polymorphism with cancer susceptibility:** Analyses of 81 case-control studies including 19547 cases and 23935 controls were conducted to explore the relationship between −511C/T and cancer risk (Table 1). Overall, no significant association was detected in any genetic model (homozygote comparison: OR = 1.09, 95% CI: 0.96–1.23; dominant model: OR = 1.04, 95% CI: 0.95–1.13). The pooled estimates remained stable when restricted to studies not deviating from HWE. Intriguingly, in the subgroup analysis stratified by cancer type, the IL-1B -511T allele showed evidence of an association with increased cervical cancer risk (dominant model: OR = 1.74, 95% CI: 1.35–2.23), but demonstrated a protective role in the development of hepatocellular carcinoma (dominant model: OR = 0.68, 95% CI: 0.47–0.99). Increased risk was also observed in blood cancers (recessive model: OR = 1.19, 95% CI: 1.04–1.37).

**Table 1:** Stratification analyses of the IL-1B –511C/T polymorphism on cancer susceptibility.

Variables	n^a^	Homozygote comparison		Heterozygote comparison		Dominant model		Recessive model	
		(TT versus CC)		(CT versus CC)		(TT+CT versus CC)		(TT versus CT+CC)	
		OR (95% CI)	P^b^	OR (95% CI)	P^b^	OR (95% CI)	P^b^	OR (95% CI)	P^b^
**Total**	81	1.09(0.96–1.23)	0.000	1.02(0.94–1.11)	0.000	1.04(0.95–1.13)	0.000	1.07(0.98–1.18)	0.000
**Studies in HWE** d	67	1.10(0.97–1.26)	0.000	1.08(0.99–1.18)	0.000	1.09 (0.99–1.19)	0.000	1.05(0.95–1.15)	0.000
**Cancer types**									
Gastric cancer	40	1.18(0.96–1.44)	0.000	1.07(0.93–1.23)	0.000	1.08(0.95–1.22)	0.000	1.14(0.99–1.31)	0.000
Lung cancer	5	0.73(0.39–1.35)	0.000	0.86(0.71–1.03)	0.121^c^	0.82(0.58–1.15)	0.007	0.80(0.49–1.31)	0.000
Blood cancers	5	1.31(0.88–1.94)	0.038	1.04(0.76–1.44)	0.002	1.07(0.77–1.47)	0.001	**1.19(1.04–1.37)**	0.104^c^
Cervical cancer	4	**1.74(1.28–2.36)**	0.206^c^	**1.71(1.32–2.23)**	0.302^c^	**1.74(1.35–2.23)**	0.338^c^	1.32(0.78–2.24)	0.001
Esophageal cancer	4	1.17(0.62–2.21)	0.006	0.98(0.83–1.16)	0.680^c^	1.00(0.86–1.18)	0.330^c^	1.19(0.72–1.95)	0.009
Hepatocellular Carcinoma	4	0.67(0.34–1.34)	0.006	**0.75(0.60–0.94)**	0.187^c^	**0.68(0.47–0.99)**	0.091	0.90(0.49–1.66)	0.003
Skin cancer	3	0.96(0.71–1.28)	0.209^c^	0.98(0.81–1.19)	0.303^c^	0.97(0.81–1.17)	0.180^c^	0.96(0.73–1.27)	0.404^c^
Prostate cancer	3	0.85(0.65–1.11)	0.121^c^	0.96(0.80–1.16)	0.309^c^	0.93(0.78–1.11)	0.156^c^	0.87(0.68–1.12)	0.243^c^
Breast cancer	3	1.10(0.70–1.73)	0.040	0.97(0.82–1.15)	0.166^c^	0.99(0.74–1.33)	0.048	1.09(0.96–1.48)	0.116^c^
Others	10	0.85(0.62–1.18)	0.043	0.96(0.67–1.38)	0.000	0.95(0.68–1.32)	0.000	0.92(0.76––1.11)	0.597^c^
**Ethnicities**									
Asian	45	1.03(0.85–1.23)	0.000	0.97(0.84–1.11)	0.000	0.98(0.85–1.14)	0.000	1.05(0.93–1.18)	0.000
Caucasian	27	1.05(0.90–1.23)	0.001	1.07(0.96–1.19)	0.000	1.06(0.95–1.19)	0.000	1.06(0.89–1.16)	0.009
Others	9	1.79(0.55–3.36)	0.000	1.05(0.88–1.24)	0.203^c^	1.25(0.89–1.73)	0.003	**1.66(1.03–2.70)**	0.000
**Source of control**									
Population-based	34	1.14(0.96–1.35)	0.000	1.07(0.97–1.17)	0.000	1.08(0.97–1.19)	0.000	1.08(0.94–1.24)	0.000
Hospital-based	47	1.03(0.86–1.25)	0.000	0.98(0.84–1.14)	0.000	1.00(0.86–1.16)	0.000	1.07(0.95–1.21)	0.000

a Number of studies;

b *P-*value of Q-test for heterogeneity test;

c A Fixed-effects model was used when the *P*-value for heterogeneity test was >0.10; otherwise, a random-effects model was used;

d HWE, Hardy–Weinberg equilibrium.

The data were additionally stratified for gastric cancer in the dominant model (Table 2). The association became significant when excluded studies not in HWE (OR = 1.16, 95% CI: 1.02–1.32). Statistically significant findings were also found in population-based case-control studies (OR = 1.20, 95% CI: 1.00–1.43) but not in analysis stratified by ethnicities. When gastric carcinoma was classified according to tumor site (cardia or non-cardia) and histopathology subtypes (intestinal or diffuse/mixed), significant associations were detected in non-cardia gastric cancer (OR = 1.57, 95% CI: 1.06–2.31) and intestinal gastric cancer (OR = 1.24, 95% CI: 1.04–1.49).

**Table 2:** Stratification analyses of the IL-1B –511C/T polymorphism on gastric cancer susceptibility.

Variables	n^a^	Dominant model	
		(TT+CT versus CC)	
		OR (95% CI)	P^b^
**Total**	40	1.08(0.95–1.22)	0.000
**Studies in HWE** d	31	**1.16(1.02–1.32)**	0.000
**Ethnicities**			
Asian	24	1.01(0.87–1.19)	0.000
Caucasian	11	1.25(0.78–2.00)	0.089
Mixed	5	1.50(1.14–1.97)	0.000
**Source of control**			
Population-based	15	**1.20(1.00–1.43)**	0.000
Hospital-based	25	0.99(0.84–1.17)	0.000
**Tumor site**			
Cardia type	5	1.24(0.94–1.65)	0.942^c^
Non-cardia type	5	**1.57(1.06–2.31)**	0.003
**Histology**			
Intestinal type	11	**1.24(1.04–1.49)**	0.033
Diffuse/Mixed type	11	1.03(0.78–1.36)	0.003
**HP status**			
HP^e^+	13	1.14(0.85–1.51)	0.000
HP-	7	1.19(0.92–1.54)	0.581^c^

a Number of studies;

b *P-*value of Q-test for heterogeneity test;

c A Fixed-effects model was used when the *P*-value for heterogeneity test was >0.10; otherwise, a random-effects model was used;

d HWE, Hardy–Weinberg equilibrium;

e HP, *Helicobacter pylori.*

The genotype distribution of −511C/T among cases and infection-match controls was available in 20 studies that investigated gastric cancer infected by *Helicobacter pylori* (HP). However, there were no significant gene-environment interactions in this case.

**Figure 2:**
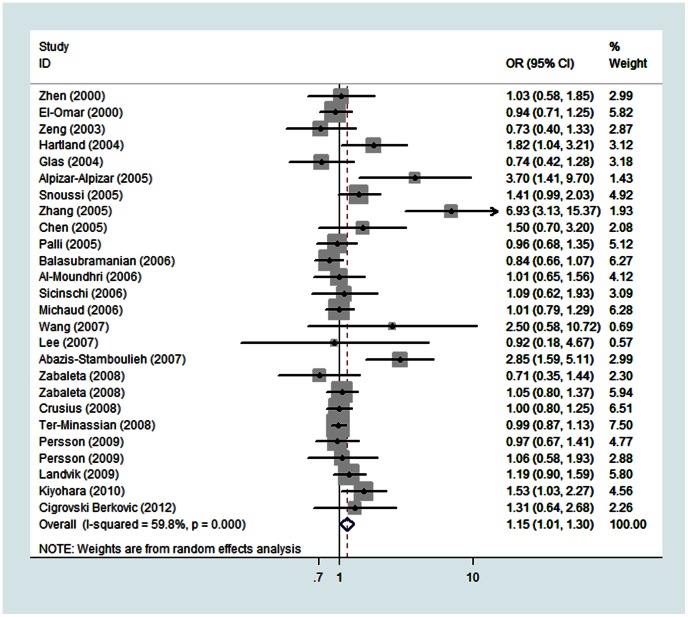
Forest plots describing the association of the IL-1B +3954C/T polymorphism with cancer susceptibility in the dominant model. The squares and horizontal lines correspond to the OR and 95% CI for each study. The area of the squares reflects the weight. The diamond represents the summary OR and 95% CI.

**Association of the IL-1B +3954C/T polymorphism with cancer susceptibility:** The analysis eventually included 26 case-control studies with 8083 cases and 9183 controls for IL-1B +3954C/T. Table 3 presents the main results of the pooled analysis and Figure 2 shows the association between +3954C/T and cancer risk in the form of forest plots. Overall, the results of combined analyses of all studies suggested that the +3954C/T polymorphism was significantly associated with increased cancer susceptibility in the dominant model (OR = 1.15, 95% CI: 1.01–1.30). This association remained consistently strong when limited to studies in HWE (heterozygote comparison: OR = 1.14, 95% CI: 1.00–1.31; dominant model: OR = 1.16, 95% CI: 1.02–1.33). When stratified by source of controls, hospital-based studies exhibited significantly increased risk (dominant model: OR = 1.30, 95% CI: 1.02–1.66).

**Table 3:** Stratification analyses of the IL-1B +3954C/T polymorphism on cancer susceptibility.

Variables	n^a^	Homozygote comparison		Heterozygote comparison		Dominant model		Recessive model	
		(TT versus CC)		(CT versus CC)		(TT+CT versus CC)		(TT versus CT+TT)	
		OR (95% CI)	P^b^	OR (95% CI)	P^b^	OR (95% CI)	P^b^	OR (95% CI)	P^b^
**Total**	26	1.13(0.97–1.31)	0.163^c^	1.13(0.99–1.28)	0.001	**1.15(1.01–1.30)**	0.000	1.01(0.95–1.29)	0.362^c^
**Studies in HWE** d	24	1.14(0.98–1.33)	0.113^c^	**1.14(1.00–1.31)**	0.000	**1.16(1.02–1.33)**	0.000	1.12(0.96–1.31)	0.280^c^
**Cancer types**									
Gastric cancer	13	0.92(0.71–1.19)	0.506^c^	1.20(0.94–1.53)	0.001	1.19(0.94–1.50)	0.000	0.91(0.70–1.17)	0.539^c^
Lung cancer	4	1.16(0.89–1.51)	0.442^c^	1.05(0.93–1.18)	0.198^c^	1.06(0.95–1.19)	0.168^c^	1.14(0.88–1.48)	0.423^c^
Prostate cancer	3	1.52(0.77–1.72)	0.626^c^	0.98(0.82–1.18)	0.403^c^	1.00(0.84–1.19)	0.597^c^	1.15(0.78–1.71)	0.533^c^
Others	6	1.74(0.91–3.34)	0.048	1.25(0.88–1.75)	0.018	1.33(0.92–1.93)	0.003	**1.47(1.04–2.10)**	0.220^c^
**Ethnicities**									
Asian	6	3.01(0.97–9.32)	0.887^c^	1.66(0.85–3.24)	0.001	1.73(0.91–3.29)	0.001	2.92(0.94–9.01)	0.863^c^
Caucasian	14	1.05(0.89–1.25)	0.124^c^	1.01(0.93–1.10)	0.120^c^	1.05(0.93–1.18)	0.051	1.04(0.88–1.23)	0.335^c^
Others	6	1.33(0.94–1.88)	0.435^c^	1.12(0.84–1.90)	0.069	1.15(0.88–1.51)	0.073	1.28(0.91–1.78)	0.427^c^
**Source of control**									
Population-based	14	1.03(0.87–1.22)	0.657^c^	1.07(0.92–1.24)	0.002	1.07(0.92–1.23)	0.002	1.03(0.87–1.22)	0.601^c^
Hospital-based	12	**1.57(1.13–2.18)**	0.148^c^	1.25(0.98–1.59)	0.029	**1.30(1.02–1.66)**	0.014	**1.44(1.04–1.99)**	0.343^c^

a Number of studies;

b *P-*value of Q-test for heterogeneity test;

c A Fixed-effects model was used when the *P*-value for heterogeneity test was >0.10; otherwise, a random-effects model was used;

d HWE, Hardy–Weinberg equilibrium.

### Meta Regression

Since there was significant heterogeneity for IL-1B –511C/T in all genetic models, a univariable regression was conducted to explore the predefined possible source of heterogeneity (Table S2). We identified that it is variability in MAF of this polymorphism across different ethnicities that was a significant source of heterogeneity (dominant model:β coefficient = −1.60 (−2.67 to −0.47), P = 0.006; recessive model: β coefficient = −1.40 (−2.45 to −0.35), P = 0.010), but not cancer type or source of control. Using dominant model, we also did meta regression for +3954C/T (Table S3). However, cancer type (*P* = 0.816), MAF (*P* = 0.050) and source of control (*P* = 0.308) only explained little of the heterogeneity.

### Sensitivity Analyses and Publication Bias

**Figure 3:**
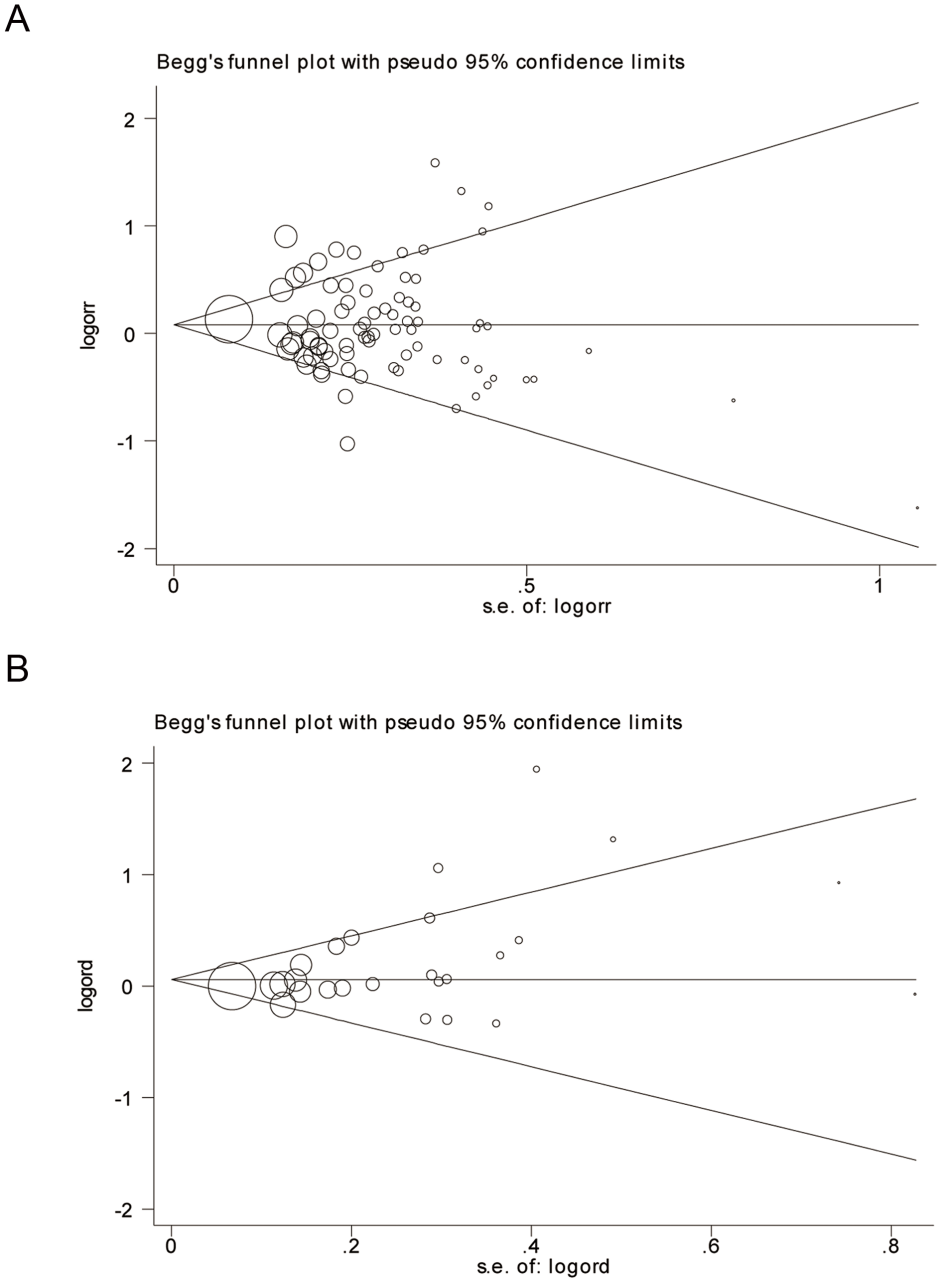
Begg’s funnel plots of publication bias. Each study is represented by a circle, the area of which presents the study’s precision. A, funnel plot for IL-1B −511C/T in the recessive model (Egger’s test P = 0.579). B, funnel plot for IL-1B +3954C/T in the dominant model (Egger’s test P = 0.020).

The influence of each study on the pooled OR was examined by repeating the meta-analyses while sequentially omitting individual studies. Both sensitivity analyses for −511C/T and +3954C/T indicated that no single study influenced the pooled ORs qualitatively, suggesting that the results of our meta-analyses are robust and stable. The shape of funnel plots was symmetrical for −511C/T, and the Egger’s test *P*-value were 0.628, 0.788, 0.888 and 0.579 for homozygote comparison, heterozygote comparison, dominant model and recessive model, respectively (Figure 3). As for the +3954C/T polymorphism, symmetrical funnel plots were found in homozygote comparison (P = 0.382) and recessive model (P = 0.509), but not in heterozygote comparison and dominant model (Egger’s test P = 0.026 and 0.020, respectively, Figure 3), implying the existence of publication bias.

The PRISMA Checklist for our study is shown as Table S4 [117].

## Discussion

Large sample epidemiological studies of predisposition gene polymorphisms could provide insight into the in vivo association between candidate genes and diseases. The present meta-analysis, including 91 high-quality case-control studies, is the most comprehensive meta-analysis to have evaluated the IL-1B polymorphisms (−511C/T and +3954C/T) and their relationship to cancer susceptibility. Its strength was based on the accumulation of published data providing greater information to detect significant differences. Our results demonstrated that IL-1B +3954C/T was significantly associated with increased overall cancer risk, especially among hospital-based case-control studies. No significant association was observed between −511C/T and overall cancer risk. However, results from subgroup analyses indicated that −511C/T was associated with susceptibility of certain types of cancer. Given the critical roles of IL-1β in inflammation and carcinogenesis, it is possible that both IL-1B −511C/T and +3954C/T polymorphisms may modulate the risk of cancer development.

Cumulative evidence suggests that IL-1β plays an important role in tumorigenesis and development. It is well known that IL-1 expression is elevated in most human cancers. High plasma IL-1β levels are associated with a significantly increased risk of cancer, and tumor patients with high IL-1 expression have worse prognosis than those without [99], [115], [118]. IL-1β promotes invasiveness, including tumor angiogenesis, and also induces immune suppression in the host [16], [17]. Moreover, IL-1β has been found to combine with estrogen receptor (ER)α in breast cancer cells, resulting in transcriptional activation [119]. In pancreatic cancer cells, IL-1β has been shown to mediate adhesion and invasion, as well as modulating chemoresistance by activating the NF- κB and ERK signaling pathways [120]–[123]. IL-1β can also attenuate interferon-induced antiviral activity and STAT1 activation in the liver, and modulate immune responses in hepatitis virus-related hepatocellular carcinoma [43], [124].

In the subgroup analysis according to cancer type, a significant association was detected between the -511T variant allele polymorphism and increased cervical cancer risk. Interestingly, this allele seemed to be a protective factor for the development of hepatocellular carcinoma. Some possible reasons may explain this discrepancy. First, the relatively small number of eligible studies in each subgroup might induce significant/insignificant association by chance due to insufficient statistical power. Second, there are some conflicting data regarding the effect of the IL-1B −511/−31 haplotype on gene expression. Ex vivo blood stimulation assay suggests that the -511T/−31C haplotype is significantly associated with an increase in LPS-induced IL-1β intracellular secretion [23]. IL-1B -511T/−31C is associated with a high level of IL-1β in the plasma [125]. However, the IL-1B -31 polymorphism involves a TATA-box motif and the -31T allele is associated with a five-fold elevated binding activity with the transcription initiation factor [19], [21], [22]. In vivo study indicates that a disease-related haplotype including −511C/−31T has higher IL-1B mRNA expression in the lungs compared with the non-risk haplotype [115]. In HP-infected gastric cancer patients, the mucosal IL-1B level is higher in −31T carriers than in −31C carriers [57]. It is likely that gene expression in each organ, such as the cervix and liver, is differently regulated. Functional study in specific tissue is required for better understanding of the role of IL-1B genotype in carcinogenesis.

Our results for gastric cancer were partly consistent with results from a previous meta-analysis performed by Xue et al. [126]. Both our meta-analysis and Xue’s demonstrated no association between IL-1B +3954C/T and gastric cancer risk. They found IL-1B −511T carriers were associated with a significantly increased risk of gastric cancer. However, we observed significant associations only among population-based studies. Our work may be interpreted as an update of Xue’s, because another 30 or more studies concerning −511C/T or +3954C/T polymorphisms and gastric susceptibility with inconsistent results have been published in the past 2 years. We also found that −511T carriers are significantly associated with risk of the non-cardia and intestinal types of gastric cancer, whereas association was not detected in HP-positive group nor in the HP-negative group, and this is consistent with Xue’s meta-analysis. Further in-depth research focused on IL-1B –511C/T and specific subtypes of gastric cancer is warranted.

In the stratification analysis of source of control, significantly increased risk between the IL-1B +3954C/T polymorphism and cancer risk was detected among hospital-based studies, but not among population-based studies. This may be due to some selection biases existing in hospital-based studies because such controls might come from a population with a related disease and may not be a representative of the general population, especially when there is a relationship between the investigated genotypes and the disease conditions that hospital-based controls might have. Although it is more convenient to recruit hospital controls, to reduce biases in such genetic association studies it may be preferable to use population controls.

Significant heterogeneity existed among studies for IL–511C/T in all comparisons, and meta regression indicated that MAF in different ethnicities partly accounts for the heterogeneity. Although we observed a wide variation of -511T allele frequency of control resources in different populations, and the MAF might reflect an environmental impact on gene distribution, subgroup analysis stratified by ethnicity did not detect any significant association between –511C/T and cancer risk in Asians nor in Caucasians. Cancer is a multi-factorial disease resulting from complex interactions between environmental and genetic factors [1]. Some other factors may weaken the effect of IL-1B –511C/T on cancer risk in different ethnic groups.

Some limitations of our meta-analysis should be mentioned. Our results were based on unadjusted estimates because of the absence of available information. If more detailed individual data such as age, sex and exposure were available, a more precise analysis would be performed. Additionally, both the asymmetric funnel plot and Egger’s test indicated the existence of publication bias in two comparisons for the IL-1B +3954C/T polymorphism. This may be explained by the restriction of the meta-analysis to studies published in English. In spite of these limitations, our meta-analysis also had some advantages. First, it is the most comprehensive meta-analysis to evaluate the two polymorphisms (−511C/T and +3954C/T) and their relationship to cancer susceptibility. The number of cases and controls through the pooled studies could significantly increase the statistical power of the analysis. Second, all included studies had acceptable quality (scored at least 5).

### Conclusions

In conclusion, this meta-analysis indicated that IL-1B +3954C/T was associated with significantly increased overall cancer risk, especially among hospital-based case-control studies. The IL-1B −511T allele showed evidence of an association with increased cervical cancer risk but demonstrated a protective role in the development of hepatocellular carcinoma. Moreover, −511C/T was associated with risk of specific subtypes of gastric carcinoma. Further large-sample research should use standardized unbiased homogeneous cancer patients and well-matched controls to confirm our findings. Additionally, gene-environment interactions should be considered in future studies.

## Supporting Information

Figure S1 **Minor allele frequencies of IL-1B −511C/T and +3954C/T polymorphisms among ethnicities of Asian, Caucasian and Others in controls.**
(TIF)

Table S1 **Main characteristics of the selected studies.**
(DOC)

Table S2 **Results of random effect meta-regression for search of source of heterogeneity for IL-1B –511C/T.**
(DOC)

Table S3 **Results of random effect meta-regression for search of source of heterogeneity for IL-1B +3954C/T in the dominant model.**
(DOC)

Table S4 **PRISMA Checklist for current meta-analysis.**
(DOC)
