# Supplementary figures and images for: Systematic Review and Meta-Analysis on the Association between IL-1B Polymorphisms and Cancer Risk
Source: PLoS One. 2013 May 21;8(5):e63654. doi: 10.1371/journal.pone.0063654 (PMC3660576; doi:10.1371/journal.pone.0063654)

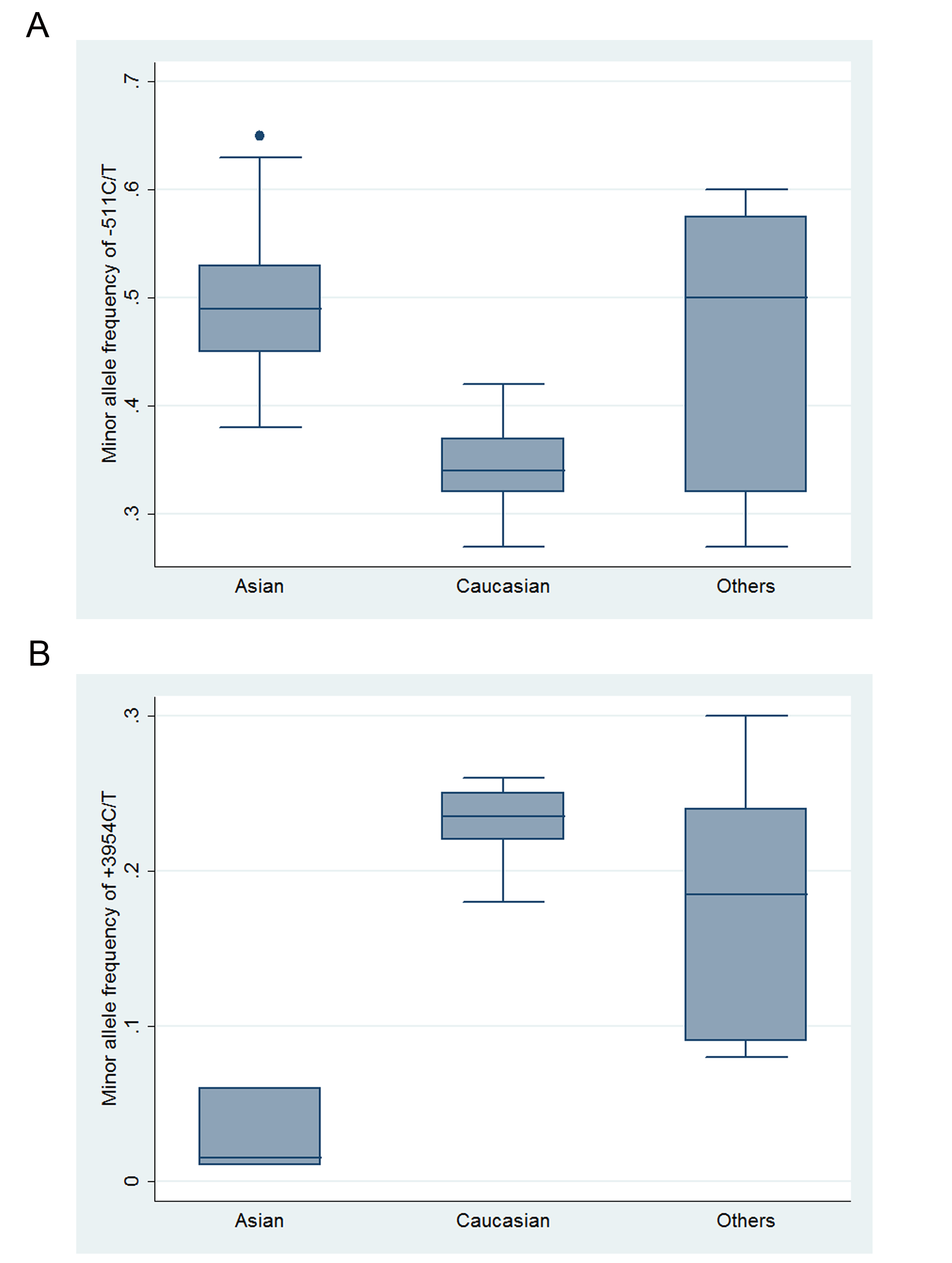

Supplement: Figure S1 — Minor allele frequencies of IL-1B −511C/T and +3954C/T polymorphisms among ethnicities of Asian, Caucasian and Others in controls. (TIF) [file pone.0063654.s001.tif]
