# Supplementary material for: Systematic Review and Meta-Analysis on the Association between IL-1B Polymorphisms and Cancer Risk
Source: PLoS One. 2013 May 21;8(5):e63654. doi: 10.1371/journal.pone.0063654 (PMC3660576; doi:10.1371/journal.pone.0063654)
Supplement: Table S1 — Main characteristics of the selected studies. (DOC) [file pone.0063654.s002.doc]

Table S1. Main characteristics of the selected studies.

| First auther | Year | Cancer type | Country | Ethnicity | SNPs studied | Control based | Genotyping method | Cases/Controls | MAF in controls | HWE value | Quality Score |
| --- | --- | --- | --- | --- | --- | --- | --- | --- | --- | --- | --- |
| Zheng [107] | 2000 | multiple myeloma | Sweden | Caucasian | +3954C/T | hospital | PCR-RFLP | 73/129 | 0.22 | 0.79 | 5 |
| El-Omar [19] | 2000 | gastric cancer | Poland | Caucasian | -511C/T; +3954C/T | population | PCR-SSCP | 366/429; 366/429 | 0.30; 0.25 | 0.10; 0.64 | 8 |
| Hwang [39] | 2002 | gastric cancer | Japan | Asian | -511C/T | hospital | PCR-RFLP | 19/117 | 0.44 | 0.4 | 7 |
| Wang [40] | 2003 | hepatocellular carcinoma | Japan | Asian | -511C/T | hospital | direct sequencing | 125/55 | 0.54 | 0.32 | 8 |
| Machado [31] | 2003 | gastric cancer | Portugal | Caucasian | -511C/T | population | PCR-SSCP | 287/306 | 0.34 | 0.27 | 7 |
| Howell [41] | 2003 | cutaneous malignant melanoma | UK | Caucasian | -511C/T | hospital | ARMS–PCR | 261/154 | 0.36 | 0.73 | 7 |
| El-Omar [38] | 2003 | esophageal cancer | USA | Mixed | -511C/T | population | TaqMan | 161/210 | 0.27 | 0.02* | 5 |
| El-Omar [38] | 2003 | gastric cancer | USA | Mixed | -511C/T | population | TaqMan | 314/210 | 0.27 | 0.02* | 5 |
| Zeng [42] | 2003 | gastric cancer | China | Asian | -511C/T; +3954C/T | hospital | PCR-RFLP | 170/362; 170/361 | 0.42; 0.06 | 0.94; 0.21 | 7 |
| Tanaka [43] | 2003 | hepatocellular carcinoma | Japan | Asian | -511C/T | hospital | PCR-RFLP | 146/230 | 0.45 | 0.02* | 6 |
| Zhang [44] | 2004 | basal cell carcinoma | Sweden | Caucasian | -511C/T | population | Pyrosequencing | 241/260 | 0.38 | 0.14 | 7 |
| Yang [45] | 2004 | gastric cancer | China | Asian | -511C/T | population | PCR-RFLP | 280/258 | 0.52 | 0.37 | 9 |
| Chen [46] | 2004 | gastric cancer | Taiwan | Asian | -511C/T | hospital | PCR-RFLP | 142/164 | 0.51 | 0.08 | 5 |
| Zienolddiny [47] | 2004 | lung cancer | Norway | Caucasian | -511C/T | population | TaqMan | 251/270 | 0.42 | 0.11 | 7 |
| Lee [48] | 2004 | gastric cancer | Korea | Asian | -511C/T | population | PCR-RFLP | 331/433 | 0.54 | 0.49 | 5 |
| Wu [37] | 2004 | gastric cancer | China | Asian | -511C/T | population | PCR-SSOP | 204/210 | 0.45 | 0.10 | 8 |
| Wu [37] | 2004 | maltoma | China | Asian | -511C/T | population | PCR-SSOP | 70/210 | 0.45 | 0.10 | 8 |
| Grimm [49] | 2004 | vulvar carcinogenesis | Austria | Caucasian | -511C/T | hospital | PCR, Pyrosequencing | 65/227 | 0.37 | 0.34 | 5 |
| Hartland [52] | 2004 | gastric cancer | UK | Caucasian | -511C/T; +3954C/T | hospital | PCR-RFLP | 59/287; 59/286 | 0.42; 0.21 | <0.01*; 0.62 | 6 |
| Glas [51] | 2004 | gastric cancer | Germany | Caucasian | -511C/T; +3954C/T | population | PCR-RFLP | 88/145; 88/145 | 0.35; 0.22 | 0.14; 0.37 | 8 |
| Tsai [52] | 2005 | bladder cancer | China | Asian | -511C/T | population | PCR-RFLP | 123/105 | 0.5 | 0.63 | 5 |
| Sakuma [53] | 2005 | gastric cancer | China | Asian | -511C/T | hospital | PCR-RFLP | 140/103 | 0.49 | 0.37 | 8 |
| Alpizar-Alpizar [54] | 2005 | gastric cancer | Costa Rica | Mixed | -511C/T; +3954C/T | hospital | PCR-RFLP | 50/50; 45/45 | 0.57; 0.09 | 0.66; 0.51 | 8 |
| Snoussi [108] | 2005 | breast cancer | Tunisia | African | +3954C/T | hospital | PCR-RFLP | 305/200 | 0.24 | 0.25 | 5 |
| Perri [55] | 2005 | gastric cancer | Italy | Caucasian | -511C/T | population | DHPLC | 184/362 | 0.34 | 0.98 | 6 |
| Zhang [56] | 2005 | gastric cancer | China | Asian | -511C/T; +3954C/T | population | PCR-RFLP | 154/166;154/166 | 0.53; 0.02 | 0.07; 0.75 | 7 |
| Chang [57] | 2005 | gastric cancer | South Korea | Asian | -511C/T | hospital | PCR-RFLP | 234/434 | 0.52 | 0.01* | 6 |
| Lu [58] | 2005 | gastric cancer | China | Asian | -511C/T | population | DHPLC | 250/300 | 0.51 | 0.13 | 9 |
| Ruzzo [59] | 2005 | gastric cancer | Italy | Caucasian | -511C/T | population | PCR-RFLP | 138/100 | 0.31 | 0.22 | 8 |
| Tsai [60] | 2005 | oral cancer | China | Asian | -511C/T | hospital | PCR-RFLP | 130/105 | 0.5 | 0.63 | 6 |
| Chen [61] | 2005 | hepatocellular carcinoma | China | Asian | -511C/T; +3954C/T | population | PCR-RFLP | 573/384; 573/385 | 0.45; 0.01 | 0.76; 0.80 | 8 |
| Palli [109] | 2005 | gastric cancer | Italy | Caucasian | +3954C/T | population | RFLP+Real time | 185/546 | 0.23 | 0.24 | 6 |
| Taguchi [62] | 2005 | gastric cancer | Japan | Asian | -511C/T | hospital | TaqMan | 373/250 | 0.46 | 0.27 | 8 |
| Hefler [63] | 2005 | breast cancer | Germany, Austria | Caucasian | -511C/T | hospital | PCR, Pyrosequencing | 269/227 | 0.37 | 0.44 | 8 |
| Asada [64] | 2006 | lung cancer | Japan | Asian | -511C/T | population | PCR-RFLP | 220/224 | 0.57 | 0.09 | 8 |
| Al-Moundhri [110] | 2006 | gastric cancer | Oman | Mixed | +3954C/T | population | TaqMan | 118/245 | 0.3 | 0.09 | 6 |
| Balasubramanian [65] | 2006 | breast cancer | UK | Caucasian | -511C/T; +3954C/T | population | TaqMan | 703/489; 691/420 | 0.31; 0.25 | 0.60; 0.24 | 7 |
| Kim [66] | 2006 | gastric cancer | Korea | Asian | -511C/T | population | PCR-RFLP | 237/474 | 0.55 | 0.02* | 9 |
| Kamangar [67] | 2006 | gastric cancer | Finland | Caucasian | -511C/T | population | TaqMan | 104/165 | 0.38 | 0.01* | 7 |
| Starzynska [68] | 2006 | gastric cancer | Poland | Caucasian | -511C/T | hospital | PEA | 121/119 | 0.27 | 0.06 | 8 |
| Rothman [69] | 2006 | non-Hodgkin lymphoma | Italy,Spain, Germany, USA, Canada, and US | Caucasian | -511C/T | population | Taqman, Pyrosequencing, allele-specific PCR | 3067/3490 | 0.33 | 0.33 | 7 |
| Sicinschi [111] | 2006 | gastric cancer | USA | Mixed | +3954C/T | hospital | TaqMan | 137/262 | 0.08 | 0.07 | 7 |
| Hirankarn [70] | 2006 | hepatocellular carcinoma | Thailand | Asian | -511C/T | hospital | PCR-SSP | 46/152 | 0.54 | 0.58 | 6 |
| Ikehara [71] | 2006 | gastric cancer | Japan | Asian | -511C/T | hospital | CTPP | 270/267 | 0.45 | 0.26 | 7 |
| Yamada [72] | 2006 | gastric cancer | Thailand | Asian | -511C/T | hospital | PCR-RFLP | 52/109 | 0.57 | 0.92 | 6 |
| Liu [73] | 2006 | breast cancer | China | Asian | -511C/T | population | PCR-RFLP | 365/631 | 0.45 | 0.58 | 8 |
| Michaud [74] | 2006 | prostate cancer | USA | Mixed | -511C/T; +3954C/T | population | TaqMan | 473/607; 486/614 | 0.37; 0.22 | 0.51; 0.67 | 9 |
| Ito [35] | 2007 | esophageal cancer | Japan | Asian | -511C/T | hospital | PCR-SSCP | 75/136 | 0.53 | 0.04* | 6 |
| Ito [35] | 2007 | gastric cancer | Japan | Asian | -511C/T | hospital | PCR-SSCP | 156/136 | 0.53 | 0.04* | 6 |
| Ito [35] | 2007 | colorectal cancer | Japan | Asian | -511C/T | hospital | PCR-SSCP | 70/136 | 0.53 | 0.04* | 6 |
| Sugimoto [75] | 2007 | gastric cancer | Japan | Asian | -511C/T | hospital | PCR-RFLP | 105/172 | 0.49 | 0.54 | 8 |
| Kang [76] | 2007 | cervical cancer | Korea | Asian | -511C/T | hospital | PCR-RFLP | 182/364 | 0.57 | 0.11 | 6 |
| Wang [112] | 2007 | gastric cancer | China | Asian | +3954C/T | hospital | ALM-ASA | 97/141 | 0.01 | 0.90 | 5 |
| Sun [77] | 2007 | gastric cancer | China | Asian | -511C/T | hospital | Oligochip | 65/65 | 0.44 | 0.02* | 5 |
| Seno [78] | 2007 | gastric cancer | Japan | Asian | -511C/T | hospital | PCR-RFLP | 99/93 | 0.44 | 0.42 | 6 |
| Zumkeller [79] | 2007 | colorectal cancer | Germany | Caucasian | -511C/T | population | PCR-RFLP | 364/459 | 0.34 | 0.89 | 7 |
| Li [80] | 2007 | gastric cancer | China | Asian | -511C/T | hospital | PCR-RFLP | 143/257 | 0.46 | 0.24 | 6 |
| Lee [81] | 2007 | lung cancer | China | Asian | -511C/T; +3954C/T | population | Real-time | 117/111; 119/110 | 0.40; 0.01 | 0.65; 0.88 | 7 |
| Abazis-Stamboulieh [82] | 2007 | multiple myeloma | Greece | Caucasian | -511C/T; +3954C/T | hospital | PCR-SSP | 74/160; 74/160 | 0.32; 0.24 | 0.53; 0.52 | 6 |
| Ennas [83] | 2008 | chronic lymphocytic leukaemia | Italy | Caucasian | -511C/T | population | TaqMan | 40/112 | 0.33 | 0.63 | 8 |
| Hoeft [84] | 2008 | lymphoma | Germany | Caucasian | -511C/T | population | Pyrosequencing, TaqMan, PCR-RFLP | 658/658 | 0.31 | 0.05 | 6 |
| Upadhyay [85] | 2008 | esophageal cancer | India | Asian | -511C/T | hospital | PCR-RFLP | 159/194 | 0.63 | 0.76 | 7 |
| Zhu [86] | 2008 | nasopharyngeal carcinoma | China | Asian | -511C/T | hospital | PCR-RFLP | 113/144 | 0.48 | 0.10 | 7 |
| Zabaleta [36] | 2008 | prostate cancer | USA | Caucasian | -511C/T; +3954C/T | hospital | TaqMan | 477/394; 470/389 | 0.35; 0.24 | 0.33; 0.88 | 8 |
| Zabaleta [36] | 2008 | prostate cancer | USA | African | -511C/T; +3954C/T | hospital | TaqMan | 67/129; 67/125 | 0.50; 0.15 | 0.09*; 0.85 | 8 |
| Vishnoi [87] | 2008 | gallbladder cancer | India | Asian | -511C/T | hospital | PCR-RFLP | 124/166 | 0.65 | 0.04* | 6 |
| Singh [88] | 2008 | cervical cancer | India | Asian | -511C/T | hospital | PCR-RFLP | 150/162 | 0.42 | 0.06 | 7 |
| Shin [89] | 2008 | gastric cancer | Korea | Asian | -511C/T | hospital | PCR-RFLP | 122/100 | 0.54 | 0.04* | 8 |
| Crusius [113] | 2008 | gastric cancer | Netherlands | Caucasian | +3954C/T | population | Real-time | 428/1125 | 0.25 | 0.71 | 7 |
| Ter-Minassian [114] | 2008 | lung cancer | USA | Caucasian | +3954C/T | population | Taqman | 2150/1492 | 0.23 | 0.12 | 6 |
| Hamacher [90] | 2009 | pancreatic cancer | Germany | Caucasian | -511C/T | hospital | PCR-RFLP | 73/235 | 0.33 | 0.79 | 6 |
| Ahirwar [91] | 2009 | bladder cancer | India | Asian | -511C/T | hospital | PCR-RFLP | 213/287 | 0.42 | 0.30 | 8 |
| Persson [34] | 2009 | gastric cancer | Sweden | Caucasian | -511C/T; +3954C/T | population | Seminested | 285/241; 284/242 | 0.34; 0.18 | 0.91; <0.01* | 7 |
| Persson [34] | 2009 | gastric cancer | Sweden | Caucasian | -511C/T; +3954C/T | hospital | Seminested | 65/297;65/297 | 0.39; 0.18 | 0.51; 0.01* | 7 |
| Kumar [92] | 2009 | gastric cancer | India | Asian | -511C/T | hospital | PCR-RFLP | 136/110 | 0.48 | 0.79 | 5 |
| Gehmert [93] | 2009 | gastric cancer | Peru | Mixed | -511C/T | hospital | PCR | 133/70 | 0.58 | <0.01* | 7 |
| Landvik [115] | 2009 | lung cancer | Norway | Caucasian | +3954C/T | population | TaqMan | 357/430 | 0.24 | 0.12 | 8 |
| Melo Barbosa [94] | 2009 | gastric cancer | Brazil | Mixed | -511C/T | hospital | PCR-RFLP | 30/100 | 0.46 | 0.46 | 7 |
| Whiteman [95] | 2010 | esophageal cancer | Australia | Caucasian | -511C/T | population | Sequenom iPLEX | 703/1297 | 0.34 | 0.46 | 8 |
| Kiyohara [116] | 2010 | lung cancer | Japan | Asian | +3954C/T | population | TaqMan | 462/379 | 0.06 | 0.20 | 7 |
| Wex [96] | 2010 | gastric cancer | Germany | Caucasian | -511C/T | hospital | PCR-RFLP | 116/94 | 0.32 | 0.88 | 5 |
| Yu [97] | 2010 | gastric cancer | China | Asian | -511C/T | population | PCR-RFLP | 501/500 | 0.38 | 0.11 | 7 |
| Yoo [98] | 2010 | gastric cancer | Korea | Asian | -511C/T | hospital | TaqMan | 78/111 | 0.55 | 0.13 | 5 |
| Qian [99] | 2010 | cervical cancer | China | Asian | -511C/T | population | PCR-RFLP | 404/404 | 0.44 | 0.79 | 9 |
| Wu [100] | 2010 | lung cancer | China | Asian | -511C/T | hospital | PCR-RFLP | 213/213 | 0.46 | 0.67 | 6 |
| Al-Tahhan [101] | 2011 | cervical cancer | Egypt | African | -511C/T | population | PCR-RFLP | 100/50 | 0.6 | 0.56 | 7 |
| Lim [102] | 2011 | lung cancer | Singapore | Asian | -511C/T | population | TaqMan | 299/716 | 0.47 | 0.86 | 8 |
| Rizzato [103] | 2011 | basal cell carcinoma | Hungary, Romania and Slovakia | Caucasian | -511C/T | hospital | allele-specific PCR | 492/506 | 0.35 | 0.55 | 7 |
| He [104] | 2011 | gastric cancer | China | Asian | -511C/T | hospital | PCR-RFLP | 392/508 | 0.45 | 0.18 | 5 |
| Cigrovski Berkovic [105] | 2012 | pancreatic neuroendocrine Tumors | Croatia | Caucasian | -511C/T; +3954C/T | hospital | TaqMan | 60/60; 60/60 | 0.31; 0.26 | 0.43; 0.50 | 5 |
| Santos [106] | 2012 | gastric cancer | Brazil | Mixed | -511C/T | hospital | PCR-RFLP | 64/138 | 0.54 | 0.56 | 6 |

MAF, minor allele frequency; HWE, Hardy–Weinberg equilibrium; PCR, polymerase chain reaction; RFLP, restriction fragment length polymorphisms; TaqMan, TaqMan allelic discrimination test (5' fluorogenic nuclease PCR assays); SSCP, Single strand conformation polymorphism; ARMS, amplification refractory mutation system; SSOP, sequence-specific oligonucleotide probe; DHPLC, PCR-based denaturing high-performance liquid chromatography; Real-time, Real-time PCR; PEA, primer extension assay; SSP, sequence specific primer; CTPP, Conforming two-pair primers; ALM-ASA, adapter ligation-mediated allele-specific amplification; Seminested: Seminested polymerase chain reaction.

* Controls in Hardy–Weinberg equilibrium: P <0.05.
