# Supplementary material for: Systematic Review and Meta-Analysis on the Association between IL-1B Polymorphisms and Cancer Risk
Source: PLoS One. 2013 May 21;8(5):e63654. doi: 10.1371/journal.pone.0063654 (PMC3660576; doi:10.1371/journal.pone.0063654)
Supplement: Table S2 — Results of random effect meta-regression for search of source of heterogeneity for IL-1B –511C/T. (DOC) [file pone.0063654.s003.doc]

Table S2. Results of random effect meta-regression for search of source of heterogeneity for IL-1B -511C/T.

| Possible source of heterogeneity | β coefficient (95%CI) | P-value | τ^2^ |
| --- | --- | --- | --- |
| **Cancer type** |  |  |  |
| TT vs. CC | 0.02(-0.04-0.09) | 0.446 | 0.258 |
| CT vs. CC | 0.03(-0.02-0.07) | 0.252 | 0.114 |
| TT+CT vs. CC | 0.03 (-0.02-0.08) | 0.218 | 0.141 |
| TT vs. CT+CC | 0.00 (-0.04-0.05) | 0.884 | 0.110 |
| **MAF** |  |  |  |
| TT vs. CC | **-2.35(-3.80--0.90)** | **0.002** | **0.210** |
| CT vs. CC | **-1.35(-2.42--0.29)** | **0.013** | **0.102** |
| TT+CT vs. CC | **-1.60(-2.67--0.47)** | **0.006** | **0.120** |
| TT vs. CT+CC | **-1.40(-2.45--0.35)** | **0.010** | **0.092** |
| **Source of control** |  |  |  |
| TT vs. CC | 0.10(-0.18-0.38) | 0.473 | 0.257 |
| CT vs. CC | 0.08(-0.18-0.28) | 0.418 | 0.114 |
| TT+CT vs. CC | -0.07 (-0.14-0.28) | 0.497 | 0.141 |
| TT vs. CT+CC | 0.01 (-0.19-0.21) | 0.923 | 0.110 |

MAF, minor allele frequency.
