# Supplementary material for: Systematic Review and Meta-Analysis on the Association between IL-1B Polymorphisms and Cancer Risk
Source: PLoS One. 2013 May 21;8(5):e63654. doi: 10.1371/journal.pone.0063654 (PMC3660576; doi:10.1371/journal.pone.0063654)
Supplement: Table S3 — Results of random effect meta-regression for search of source of heterogeneity for IL-1B +3954C/T in the dominant model. (DOC) [file pone.0063654.s004.doc]

Table S3. Results of random effect meta-regression for search of source of heterogeneity for IL-1B +3954C/T in the dominant model.

| Possible source of heterogeneity | TT+CT vs. CC | | |
| --- | --- | --- | --- |
|  | β coefficient (95%CI) | P-value | τ^2^ |
| Cancer type | 0.02 (-0.16-0.20) | 0.816 | 0.084 |
| MAF | -2.09(-4.12-0.003) | 0.050 | 0.043 |
| Source of control | -0.18 (-0.53-0.17) | 0.308 | 0.059 |

MAF, minor allele frequency.
