# Supplementary material for: Systematic Review and Meta-Analysis on the Association between IL-1B Polymorphisms and Cancer Risk
Source: PLoS One. 2013 May 21;8(5):e63654. doi: 10.1371/journal.pone.0063654 (PMC3660576; doi:10.1371/journal.pone.0063654)
Supplement: Table S4 — PRISMA Checklist for current meta-analysis. (DOC) [file pone.0063654.s005.doc]

| **Section/topic**  Table S4. PRISMA Checklist for current meta-analysis. | **#** | **Checklist item** | **Reported on page #** |
| --- | --- | --- | --- |
| **TITLE** | | |  |
| Title | 1 | Systematic review and meta-analysis on the association between IL-1B polymorphisms and cancer risk | 1 |
| **ABSTRACT** | | |  |
| Structured summary | 2 | Background: Interleukin-1 beta (IL-1β), a pro-inflammatory cytokine, is emerging as a key mediator of carcinogenesis that characterize host-environment interactions. Epidemiological studies investigating the association between two polymorphisms of IL-1B (-511C/T and +3954C/T) and cancer susceptibility showed conflicting results. The aim of this study is to derive a more precise estimation of the relationship.  Methods: A systemic literature search of PubMed and Web of Science was conducted from their inception to September 15, 2012, to retrieve related studies. Summary odds rations (ORs) and 95% confidence intervals (CIs) for IL-1B -511C/T and +3954C/T polymorphisms and cancer risk were calculated. Heterogeneity among studies and publication bias were also tested.  Results: A total of 91 case-control studies in 85 publications, of which 81 studies for -511C/T (19547 cases and 23935 controls) and 26 studies for +3954C/T polymorphisms (8083 cases and 9183), were included in the meta-analysis. The pooled results indicated that IL-1B +3954C/T (dominant model: OR=1.15, 95%CI: 1.01-1.30), especially among hospital-based case-control studies (dominant model: OR=1.30, 95%CI: 1.02-1.66). As for -511C/T, we observed an inverse relationship in cervical caner (dominant model: OR=1.74, 95%CI: 1.35-2.23) and hepatocelluar carcinoma (dominant model: OR=0.68, 95%CI: 0.47-0.99). Moreover, the -511C/T was associated with risk of specific subtypes of gastric carcinoma.  Conclusion: This meta-analysis suggested that both IL-1B -511C/T and +3954C/T polymorphisms might modulate cancer susceptibility. More well-designed studies based on larger sample size should be performed to confirm the findings. | 2 |
| **INTRODUCTION** | | |  |
| Rationale | 3 | There is an increasing recognition that inflammation contribute to pathogenesis of many cancers. Production of cytokines by inflammatory/immune cells is a major mechanism promoting carcinogenesis. Cytokines are glycoproteins or soluble proteins which act as mediators of inflammatory response and are integral to the function of immune cells. The role of cytokines in cancer immunity and carcinogenesis in general has been well established [9]. Interleukin-1 (IL-1) is a pro-inflammatory cytokine with multiple biological effects. IL-1β is emerging as a key mediator of carcinogenesis that characterize host-environment interactions.  The IL-1B gene is highly polymorphic and base transitions between C and T at positions -511 (C-T; dbSNP: rs16944), -31 (T-C; dbSNP: rs1143627) and +3954 (C-T; dbSNP: rs1143634) bp from the transcriptional site have been widely reported. Recently, a meta-analysis of IL-1B -31T/C polymorphism and cancer risk has suggested that the -31C allele is a low-penetrance protective factor for the development of cancer [26]. Furthermore, numerous epidemiological studies have investigated the association between IL-1B -511C/T and +3954C/T and different cancers, such as gastric, lung and breast cancers. However, the results remain inconsistent and inconclusive. A clearer understanding of the relationship between these two SNP and cancer susceptibility is of clinical significance. | 3 |
| Objectives | 4 | We did a comprehensive meta-analysis to derive a precise estimation of the relationship between IL-1B (-511C/T and +3954C/T) and cancer susceptibility. A systemic literature search of PubMed and Web of Science was conducted. Two investigators extracted information from all eligible publications independently according to the inclusion and exclusion criteria. Summary odds rations (ORs) and 95% confidence intervals (CIs) for IL-1B -511C/T and +3954C/T polymorphisms and cancer risk were calculated. Our meta-analysis suggested that both IL-1B -511C/T and +3954C/T polymorphisms might modulate cancer susceptibility. | 3-4 |
| **METHODS** | | |  |
| Protocol and registration | 5 | No review protocol exists. |  |
| Eligibility criteria | 6 | Eligible studies were selected according to the following explicit inclusion criteria: (i) case-control study evaluated the association between at least one of these two polymorphisms (IL-1B -511C/T and +3954C/T) and cancer susceptibility, (ii) sufficient genotype data presented to calculated the odds ratios (ORs) and 95% confidence intervals (CIs). Major reasons for exclusion of studies were: (i) only cancer group, (ii) no usable genotype frequency data, (iii) duplicate of earlier publication, (iv) articles not published in English. | 4 |
| Information sources | 7 | Electronic databases PubMed and Web of Science were used. The last search update on September 15, 2012. | 4 |
| Search | 8 | We searched electronic databases (PubMed and Web of Science) using combinations of the terms “interleuk-1/IL-1” or “Interleuk-1B/IL-1B”, “polymorphism” and “cancer” or “tumor” (the last search update on September 15, 2012). For each identified study, additional studies were sought from its citations, references and from the database option “Related Articles”. | 4 |
| Study selection | 9 | Case-control study met the inclusion criteria was included. Details of the literature search process are presented in the flow chart (Figure 1). | Figure 1 |
| Data collection process | 10 | Two investigators independently extracted the following information from each study: All item-specific ambiguities were discussed by investigators’ consultation until consensus was achieved. | 4 |
| Data items | 11 | The following items were extracted from each study: the first author’s name, year of publication, cancer type, country of origin, ethnicity, source of control, genotyping method, number of cases and controls and genotype frequency. Hardy–Weinberg equilibrium (HWE) in the controls was calculated for each included study using goodness-of-fit test. | 4 |
| Risk of bias in individual studies | 12 | We used the Newcastle-Ottawa quality assessment scale (NOS) to assess the quality of each study. Studies with a score equal to or higher than 5 were considered “high-quality”, whereas those less than 4 were considered “low-quality”. Two case-control studies were considered “low-quality” (score = 3 and 4, respectively) using the NOS quality assessment instrument and thus were excluded. | 4-5  “Materials and Methods” section and “Results” section |
| Summary measures | 13 | Summary odds ratios (ORs) with 95% confidence intervals (CIs) were calculated for pooled analyses. | 4 |
| Synthesis of results | 14 | The strength of the association between the two SNPs (IL-1B -511C/T and +3954C/T) and cancer risk was measured by ORs with 95%CIs. Pooled ORs were obtained from combination of single studies by homozygote comparison (TT vs. CC), heterozygote comparison (CT vs. CC), dominant and recessive models (TT+CT vs. CC, CC+CT vs. TT), respectively. The heterogeneity among different studies was checked by the Q-test. | 4 |

Page 1 of 2

| **Section/topic** | **#** | **Checklist item** | **Reported on page #** |
| --- | --- | --- | --- |
| Risk of bias across studies | 15 | Publication bias was diagnosed with Begg’s funnel plot and Egger’s linear regression method. Asymmetric or incomplete funnel shaped plots and the P-value less than 0.05 in Egger’s test indicated the presence of potential publication bias. | 5 |
| Additional analyses | 16 | Sensitivity analysis was performed to assess the stability of the results by omitting a single study in this meta-analysis each time to reflect the influence of individual data to the pooled OR. To further explore the potential source of heterogeneity among studies and test the effects of study characteristics on the overall estimates, subgroup analyses and meta regression were performed stratified by cancer types, ethnicity, source of controls, study sample size. | 4 |
| **RESULTS** | | |  |
| Study selection | 17 | Details of the literature search process are presented in the flow chart (Figure 1) and the “Flow of include studies” section. | Figure 1 and page 5 |
| Study characteristics | 18 | Detailed characteristics of the aggregated data for 91 case-control studies are summarized in Table S1 and the “study characteristics” section. | Table S1 and page 5 |
| Risk of bias within studies | 19 | We used the Newcastle-Ottawa quality assessment scale (NOS) to assess the quality of each study. Studies with a score equal to or higher than 5 were considered “high-quality”, whereas those less than 4 were considered “low-quality”. Two case-control studies were considered “low-quality” (score = 3 and 4, respectively) using the NOS quality assessment instrument and thus were excluded. | 4-5  “Materials and Methods” section and “Results” section |
| Results of individual studies | 20 | Forest plot for IL-1B +3954C/T was presented as Figure 2. | Figure 2 |
| Synthesis of results | 21 | Details of synthesis of results were presented in Table 1-3 | 19-22 |
| Risk of bias across studies | 22 | Publication bias for both polymorphisms was presented in Figure 3. | Figure 3. |
| Additional analysis | 23 | Both sensitivity analyses for -511C/T and +3954C/T indicated that no single study influenced the pooled ORs qualitatively.  The results of meta regression were presented in Table S2, S3. Variability in minor allele frequency was found to be a significant source of heterogeneity for -511C/T. | Page 6 and Table S2, S3 |
| **DISCUSSION** | | |  |
| Summary of evidence | 24 | The present meta-analysis, including 91 high-quality case-control studies, is the most comprehensive meta-analysis that evaluated the IL-1B polymorphisms (-511C/T and +3954C/T) and the relationship to cancer susceptibility. Its strength was based on the accumulation of published data providing greater information to detect significant differences. Our results demonstrated that IL-1B +3954C/T was significantly associated with increased overall cancer risk, especially among hospital-based case-control studies. No significant association was observed between -511C/T and overall cancer risk. However, results from subgroup analyses indicated that the -511C/T was associated with susceptibility of certain types of cancer. Given the critical roles of IL-1βin inflammation and carcinogenesis, it is possible that both IL-1B -511C/T and +3954C/T polymorphisms may modulate the risk of cancer development. | 7-8 |
| Limitations | 25 | Our results were based on unadjusted estimates due to the absence of available information. If more detailed individual data such as age, sex and exposure were available, a more precise analysis would be performed. Additionally, both the asymmetric funnel plot and Egger’s test indicated the existence of publication bias in two comparisons for IL-1B +3954C/T polymorphism. This may be because that only published English studies were included in the meta-analysis. | 8 |
| Conclusions | 26 | In conclusion, both IL-1B -511C/T and +3954C/T polymorphisms might modulate cancer susceptibility. More well-designed studies based on larger sample size should be performed to confirm the findings. | 8-9 |
| **FUNDING** | | |  |
| Funding | 27 | This study was supported by the National Natural Science Foundation of China (No. 81071643). |  |

*From:*  Moher D, Liberati A, Tetzlaff J, Altman DG, The PRISMA Group (2009). Preferred Reporting Items for Systematic Reviews and Meta-Analyses: The PRISMA Statement. PLoS Med 6(6): e1000097. doi:10.1371/journal.pmed1000097

For more information, visit: **www.prisma-statement.org**.

Page 2 of 2
